# Topics of Public Interest

**Published:** 1913-10

**Authors:** 


### TOPICS OF PUBLIC INTEREST

### Resolutions

Offered to the Fourth International Congress of School Hygiene at Buffalo, August, 1913, by S. Adolphus Knopf, M. D., N. Y.

*Whereas,* Nearly a million tuberculous children, or children strongly predisposed to tuberculosis, are attending our public schools, and there is hardly accommodation for 1500 to receive instructions in the open air; and,

*Whereas,* The Congress is convinced that the open air school is one of the most powerful agents in the prevention and cure of tuberculosis in childhood, and it has been furthermore demonstrated that nearly all climatic conditions, providing the air is dust-free, lend themselves to the prevention of tuberculosis in the predisposed and the cure of the afflicted; and,

*Whereas,* Statistics show that there are not nearly enough hospital and sanitorium accommodations for adults and children afflicted with pulmonary tuberculosis or children suffering with tuberculous joint or bone diseases; and,

*Whereas,* It has been demonstrated in New York and other cities that discarded vessels lend themselves admirably to transformation into all-year-round hospitals and sanatoria for consumptive adults, sanatoria for children afflicted with joint and other types of tuberculosis, and into open air schools for tuberculous, anemic and nervous children;

*Resolved,* That the Fourth International Congress on School Hygiene petitions the United States Government to place at the disposal of the various States of the Union as many of the discarded battle ships and cruisers as possible to be anchored according to their size in rivers or at the seashore and to be utilized by the respective communities for open-air schools, sanatorium schools for children, or hospital-sanatoria for adults. Be it further

*Resolved,* That the Congress expresses its appreciation to the Italian Government of the example it has given by consecrating three of its discarded men-of-war to the combat of tuberculosis. Be it further

*Resolved,* That this Congress expresses the sincere wish that other governments may follow the example of Italy; and be it finally

*Resolved,* That copies of these resolutions be presented to the American and other governments represented at this Congress.

School For Health Officers, Conducted by Harvard University and the Massachusetts Institute of Technology. Beginning this fall, Harvard University and the Massachusetts Institute of Technology are to maintain in co-operation a School for Public Health Officers; will confer a Certificate of Public Health (C. P. H.), signed by both President Lowell and President Maclaurin. The object of this school is to prepare young men for public health work. Graduates of colleges, or technical and scientific schools, who have received adequate instruction in Physics, Chemistry, Biology and French or German, may be admitted. The medical degree is strongly urged, but not required. The Administrative Board consists of Prof. William T. Sedgwick of the M. I. T.; Prof. Milton J. Rosenau and Prof. George C. Whipple of Harvard. Prof. Rosenau is director.

---

Cocaine. The U. S. Dept. of Agriculture requires a formal statement, on blanks furnished on application, from all importers and dealers in cocaine or coca derivatives, setting forth various details, declaring that the drug is to be used in good faith. Both the national and the state governments have surrounded the use of cocaine with many troublesome though well meant restrictions which the physician should bear in mind.

---

Chief Bacteriologist. The U. S. Civil Service Commission announce a competition, closed October 6, to fill a vacancy in the Dept. of Agriculture. No formal examination will be held, but statements of qualification must be filed. An M. D. or Ph. D. degree or equivalent training and at least seven years' practical experience in bacteriologic and pathologic work will be required. It is, therefore, not surprising that the lower age limit is set at thirty, the upper at fifty. The salary is $3,500, and Uncle Sam is evidently looking for a $5,000 man.

---

Misbranding Insecticides. The U. S. Dept. of Agriculture announce a legal decision in a case against the Calvert Drug Co. of Baltimore, following the seizure of packages of "Peterman's Roach Food." Wheat flour, etc., used as bait, and obviously not possessing insecticidal properties, were not mentioned in the analysis on the label. This was construed as misbranding, though the court provided that the goods should be released on condition that the defendant gave a bond that they should not be sold until the label should be altered to show either the names and percentage amounts of all inert ingredients or else the names and percentage amounts of all ingredients having insecticide properties and the total of inert ingredients. So far as we understand the circumstances, this case involves no criminal intention on the part of the manufacturers or their dealers. While, on first thought, the general principle that every article should be sold for what it really is, receives our support, it must be borne in mind that there are a great many compounds of a more or less secret nature, which are affected potentially by this ruling. Metal and varnish polishes, cleaning compounds, lubricating oils, etc., may be mentioned. Substances of this sort support many profitable businesses and give employment, directly and indirectly, to many persons. On the other hand, providing that the compounds are really efficient, as many of them are, they are worth the price to the consumer. The decision mentioned leads logically to the destruction of all these businesses, for the moment that the formula is required to be published, extemporized formulæ will take the place of the proprietary wares. It will, of course, be argued that this helps the consumer. But, as soon as the businesses are killed, sources of information as to formulæ will be lacking, and, in the long run, counting retail prices especially for compounding, liability to errors and to use of inferior ingredients, as well as the speedily forgotten knowledge of formulæ, the consumer will be worse off than at present.

---

COURSE IN ELECTROLOGY, RADIOLOGY AND "RADIUMLOGY." Fifty lectures will be given at the Hospital of Pity, Paris, Nov. 3-Dec. 2. At the first lecture the attendants will be divided into classes of about ten and assigned to various hospitals in Paris for practical demonstrations. The entire course is free to physicians. Applicants should address either Dr. Delherm. Hopital de la Pitie, Boulevard de l'Hopital No. 83, or Dr. Aubourg, Hopital Boucicaut, Rue de la Convention, No. 62.

---

FRAUDULENT RADIO-ACTIVE WATERS. The U. S. Dept of Agriculture issues a general warning against water advertised as possessing radio-active properties. In many cases, spring water does possess radio-active properties, but, after bottling, 50 per cent. is lost in four days and 90 per cent. after 12 days. After a month, practically no radiant energy will remain. Foreign waters which do not show the radio-active properties claimed are excluded from importation. The Department is considering means to protect the public from fraudulent claims for native waters. It is interesting to observe that, in the case of radio-active waters, we now have a scientific demonstration and means of quantitation, establishing the former claims for virtues of springs not possessed for the water when bottled and used at a distance from the source. It must also be admitted that radio-activity illustrates the partial truth of certain claims of homeopathy formerly regarded as entirely imaginative. Without going to the extreme of advocating credulity toward every claim or popular notion, skeptics who argue on a priori grounds or because of the lack of scientific evidence against various theories, will occasionally be obliged to confess that they were mistaken. We speak from the standpoint of a skeptic who formerly sneered at the idea that the water itself, aside from the recreation obtained at health resorts, could have any different action at its source than when taken from a bottle, or that the natural water possessed any virtues that could not be duplicated by following its chemic formula. Having been mistaken in this regard—and in other opinions—we ask pardon if, at times, we apparently show a tendency to acknowledge the possibility of truth in what appears to be an absurdity.

---

ANOTHER CASE OF EYE INJURY FROM ACID IN GOLF BALL. September 2, Stafford Hawken, the 12-year-old son of the Assistant U. S. Attorney at Washington, had the sight of the right eye destroyed and that of the left endangered while investigating the contents of a golf ball. Several cases of this sort have already occurred, and, in view of the number of persons playing the game, it would be well if physicians would warn their acquaintances of this danger. Indeed, it is open to serious question whether the matter should not be dealt with legally, to prevent the possibility of such accidents. The danger of purely mechanic injuries from balls of all kinds is inevitable, but is comparatively slight and is sufficiently obvious to all.

---

NEW YORK COCAINE LAW—Provisions Affecting Physicians—This law regulates the sale, furnishing, disposing of, prescribing, using, giving away or posssession of alkaloid cocaine, Alpha or Beta Eucaine, or their salts; and any admixture, compound, solution or product of which either of these drugs may be an ingredient.

To furnish, give away, etc., these substances, except under conditions named hereafter, constitutes a felony.

INVENTORY.

Every duly registered, practicing physician shall make a record of the amount of each of these substances possessed by him in a book kept for the purpose. He shall specifically state in this book the amount of each kind of said substances in his possession, and the particular place in which same is kept. This book shall be open to inspection by any prosecuting officer in the State and by such persons as he may designate; and must be kept for at least five years after the date of the last entry in it.

A physician may keep on hand, at one time, not more that 1⅛ ounces of these items.

If by any means, or through any error, he finds a larger stock in his possession, it will be his duty at once, on such discovery, to notify the State Department of Health of the fact in writing, stating the amount of each substance possessed, and the place where same is kept. No more may be purchased until the amount on hand shall be reduced by lawful disposition thereof to less than 1⅛ ounces.

### Purchases.

A duly registered and qualified physician may purchase one and one-eighth ounces of these drugs in original, sealed packages, by his written order on a manufacturer or wholesale dealer in drugs, which order must be signed by himself.

Upon the delivery to him of such substances, the physician must record the purchase in a book kept for the purpose, stating date of purchase, quantity, name and form in which purchased, name and address of seller, name of purchaser, name of person making entry, a description of the package or container in which the substance is purchased; and a statement that such substance was sold and purchased in the original package, that the package was sealed, that the seals were undamaged and unbroken, that the prescribed labels were attached and were not defaced or damaged; and a statement showing how delivery was made, whether personally or by mail, express, freight or messenger.

Since wholesalers are not permitted to sell to physicians in New York State who are not duly registered and licensed to practice, all orders for cocaine preparations should have written upon them the license number of the physician ordering.

### Stock Closet.

The above record shall also show the particular place in which the substance so purchased is to be kept. This place shall not be changed without an entry being made opposite the original entry, signed by the purchaser. (Keeping in place not scheduled, a misdemeanor.) The record and statement in this book shall be signed by the purchaser.

### Dispensing.

A physician may, after a personal examination of a patient, prescribe and himself dispense any of these items to such patient, provided he executes and delivers to the patient a certificate stating the amount dispensed and the date, signed with the physician's name and address. (Failure to give certificate, a felony.)

A physician may also carry these substances for use in his profession, provided the amount so personally carried, and the amount kept in the place scheduled in his record, shall not together exceed a total of one and one-eighth ounces of such substance.

### Prescriptions.

A physician may also write prescriptions for any of these items. These prescriptions must be signed and dated by such physician.

If the total content of a prescription containing any of these substances shall exceed one grain to the fluidounce, or in the case of an ointment, two grains to the ounce, and this prescription shall be intended for use for a longer period than ten days, it is necessary for the physician to make a statement to that effect on the certificate issued by the druggist who dispenses the prescription; the patient bringing to the physician the druggist's certificate, for that purpose.

At least once in six months a physician must record, in a book kept for the purpose, the gross amount disposed of by him. If he has on hand less than the difference between the amounts purchased and the amounts disposed of, as shown by his records, it is presumed that the law has been violated. (A felony.)

### What a Druggist Does.

If, on the presentation of a physician's prescription, the druggist finds that the proportion of cocaine or its salts is greater than one per cent. of the entire amount of the prescription, he must at once call up the physician, to verify the quantity. If the proportion of cocaine or its salts be not more than one grain to the ounce, if a liquid, or not more than two grains to the ounce, if an ointment, the prescription may be repeated by the druggist, who may give the patient a copy; but, if the proportion is greater, the prescription cannot be repeated and the druggist may neither return the prescription nor a copy to the patient.

The druggist must fill out and give the patient a certificate.

---

"Temperine," a temperance drink, said to contain less than one-half per cent. of alcohol, was found by the Department of Agriculture to contain 2.77 per cent. It was legally decided that this was sufficient to be intoxicating and the manufacturer was fined.

---

Maple Syrup and Rice, advertised as high-grade products, have been shown to be of inferior grade and fines have been imposed. The Department of Agriculture is changing the meaning of "caveat emptor." The buyor is taking care, but in a systematic, official way.

NEWSPAPER APPRECIATION OF OUR PROFESSION. Last month our profession received about five full columns in the daily press of one day. Unfortunately, a couple of columns were devoted to the sad case of a young girl, killed and dismembered, on whom a physician was said to have performed an abortion. About the same space was devoted to the recrimination of a Jewish physician who claimed that Governor Sulzer had promised him the appointment as State Health Commissioner, in return for swinging the Jewish vote of New York City. Publicity of this sort injures the prestige of the entire profession.

---

TYPHOID IN NIAGARA FALLS, ONT. This city has usually been relatively free from typhoid, the division of Niagara River by Grand Island deflecting the sewage of Buffalo into the eastern channel. Over fifty cases have occurred within the last month and the engineer of the Provincial Health Board called in consultation by the local authorities, has advised a system of hypochlorite treatment.

---

BUFFALO HOSPITAL OF THE SISTERS OF CHARITY will have a new Clinic Building to cost $100,000. It will contain seven operating rooms, in tile and marble, an X-ray laboratory, pathologic laboratory, lecture hall, cooking school, etc. The corner stone was laid September 4 by Bishop Colton, assisted by Chancellor Walsh.

---

TOO MUCH EUGENICS. Pittsburgh issued 1,035 marriage licenses in August, 1912, and the number increased for several months at the rate of 10 per cent. per month. In August, 1913, after requiring a more elaborate license blank to be filled out, the number dropped to 104.

---

AUTOMOBILE CASUALTIES. Buffalo has lately decided, by conference between a committee of the Common Council and members of the Automobile Club, to forbid dazzling lights on automobiles within the city limits—without reference to the quibble attempted by some as to whether such lights were of the ordinary type of lamp or consisted in specially attached searchlights—to remove the excuse for such dangerous illumination by repairing pavements, and to make a reasonable compromise between the full stop in passing a street car at a crossing demanded by some, and the practice of plowing a track through a crowd of pedestrians. The ordinance contemplated provides that an automobile may proceed not faster than five miles an hour past a street car taking on or discharging passengers, if there is a six-foot clearance.

The dangers of automobile traffic must be seriously considered and dealt with according to wise, general principles, applicable, so far as possible, to all communities, and in a way fair to all persons concerned. We venture to point out some underlying factors on which any efficient measures of prophylaxis must depend. If it seems to any reader that such a discussion is outside the scope of a medical journal, we would remind him that the profession is logically interested in prophylaxis, not only of disease but of sudden death; that automobile accidents are, at present, one of the commonest causes of deaths of the profession itself, and that in all general considerations of the value of the medical profession to the public, the profession is held responsible for the aggregate mortality rate, at least in the negative sense that its credit for diminishing the mortality from disease is reduced by every accidental death occurring.

The introduction of the automobile has already resulted in a congestion both of urban and rural highways not contemplated when roads and streets were laid out. Washington, D. C., is almost the only city in this country in which the average street is wide enough for the adequate accommodation of street cars, standing vehicles and moving vehicles, to say nothing of pedestrians. To speak of Buffalo alone, it may be said that there are practically no direct trunk streets suitable for long distance travel, which are not occupied by car tracks, and, during the whole of the last summer, every natural route out of the city has been more or less impassible, either on account of worn out pavements, grade crossing or other improvements pursued in the most leisurely way, or what may be termed in a journal of this nature, congenital atresia, or other anomaly. If this statement is incorrect, we welcome criticism as a matter of personal information, if nothing else. Buffalo may claim to have the most system in the laying out of streets of any city in the world. As we count them, there are five beautiful and distinct systems. In parts of the city where two or three systems come together, persons born here often get lost. One can go down town from almost any part of the city by a fairly direct route, but there are large areas of the city which are practically shut off from communication with other areas, even adjacent ones, except by long detours or by dangerous and spring-damaging streets and railroad crossings. There is no street in Buffalo that runs straight with adequate width from one city line to another. The nearest approach to such an ideal is Genesee street, which is dangerously narrow for most of its length. There are only a few available streets paralleling trunk routes of street car traffic, for any considerable distance along natural routes of travel.

These criticisms are more or less applicable to all the other cities of our territory and in only one, Rochester, is there any natural obstacle (the Genesee River) affording an adequate excuse. The first radical step in preventing the disgracefully high accident and mortality rate prevalent from vehicular congestion, is to provide streets of sufficient width, properly paved, and that lead from somewhere to somewhere.

The next step is to distinguish clearly between speed and dangerous driving. Owing to the guidance of tracks, a street car should be allowed to go at any rate of speed compatible with safety in crossing, with due regard to the ability of the average eye to judge distance and speed. Owing to its constructive controlability, an automobile at twenty miles an hour is no more dangerous than a bicycle at ten or a horse drawn vehicle at eight.

The third step is to realize that statistics as to what struck such and such a number of persons, are not of much value in determining the ultimate measures required for safety. The man killed by a trolley may have had to jump for his life to avoid an automobile and the automobile may have crowded him because a motor cycle was passing to the right or because the department of streets had neglected to fill a dangerous hole in the pavement, or had filled it with a mass of sharp flints. In a long experience, including every ordinary means of conveyance, except the motor cycle, we have no hesitation in declaring that the principal danger in traffic is not the rapidly moving vehicle of any kind but the slow one, driven without regard to the rights of others. If we were the Chief of Police we would direct the patrolmen to specialize on arrests, not of automobilists nor even of those Pariahs of the highway, the motor cyclists, but of this list: vehicles of unusual width, vehicles with pipes and lumber projecting beyond the wagon box, slow moving vehicles on car tracks and that do not keep well to the curb, or that do not heed signals of passing vehicles; vehicles with drivers asleep on the seat.

A fifth cardinal principle in achieving safety from traffic accidents is a proper distribution of responsibility. The present theory is that the man driving a vehicle, and especially an automobile—the bicycle formerly held this distinction—is to blame unless he can clearly show to the contrary. Now the ultimate object of all regulations is not to place blame nor to punish offenders but to prevent injuries. In Europe, the person run down by a vehicle is subject to arrest for obstructing traffic. This idea does not appeal favorably to the average American, but, it results in a number of accidents far less than in America, in spite of a lay-out of streets 500 years instead of 100 years behind modern requirements. Somewhere between these extreme bases for traffic regulation, must be found one which shall recognize the plain physiologic facts that a man has only two eyes and that he cannot look four ways at once, and the equally plain combination of mechanic and physiologic facts that there is, as yet, no automobile made so that the various levers and steering gear and warning horn or bell can be successfully operated at once with two hands and two feet. Every few days a child or a man playing ball, or a "jay-walker" is killed in the streets. The man who ran him down may or may not be mobbed and he may or may not be found guilty criminally or be liable or not for civil damages. The main thing to consider is that some one has been unnecessarily killed. Even at the expense of fencing off the streets, providing foot bridges at street crossings, establishing more public play grounds, or of punishing parents responsible for small children or directly, those old enough to be responsible for their own safety, these accidents must be prevented and they will continue until the fact is recognized that the streets between the curbs—or kerbs, if you prefer—must be reserved, except at crossings, for vehicles of one kind or another.

The problems of street traffic have assumed an unexpectedly high degree of importance within the last few years, corresponding largely to the rapid development of the use of the horseless vehicle. In this State, there is more than one automobile to every hundred of population, and, in relation to carrying capacity, this means that about one person in twenty-five habitually does a great part of his transit through the streets in this way. This ratio is, naturally, exceeded in cities. The automobile is still highly unsatisfactory from the standpoints of economy and trouble of upkeep, in spite of statements and quasi guarantees to the contrary. There is a gradual approach toward standards of economy and durability claimed in advertisements and demanded for most other mechanic devices in common use, such as typewriters, clocks and watches, pianos, sewing machines, telephones, etc. It is perfectly possible that competition may result in a more rapid attainment of these standards and in a sudden reduction in initial cost, comparable to that observed in the case of the bicycle a few years ago, the latter due to the elimination of the "velvet" in the present gross profit of nearly 100 per cent. The achievement of either of these highly desirable results will double the magnitude of the problem of achieving safety on our streets and roads. It is high time that the problem should be attacked vigorously and sensibly.

---

FAIRCHILD BROS. & FOSTER, New York, have been awarded a gold medal for Physiological Pharmaceutical Preparations at the exhibit in connection with the International Conference of Medicine held in London in August.

---

The New Maternity Wing of the Auburn City Hospital is open. The older wooden structure forms part of the completed building, the new part being of brick. As now completed there is a reception room for friends of the expectant mother, ten single rooms, a four-bed ward, a two-bed ward, a labor room and a delivery room, two nurseries, one having an isolation room adjoining (for cases of ophthalmia, etc.) There will be separate bassinettes for each baby. The pantry and serving room are on the first floor with a dumb waiter running to the second floor. Communication between floors is by a wide stairway up which a patient may be carried on a bed if necessary, also by elevator. The floors are all of hardwood, doors inside of mahoganized birch, outside doors of oak. There is a bath on each floor and lighting is by gas and electricity. A graduate nurse in charge. Sloane maternity methods as far as practicable. Prices $18, $20 and $25 per week.

---

The University of Buffalo opened its doors on September 22, holding, for the first time, in Alumni Hall of the medical building, a general meeting of the students of all departments. We particularly commend this action, as it promotes a solidarity of university sentiment, affords opportunity for broadening acquaintance and tends to do away with the mutual contempt which was formerly so characteristic of the departments of universities elsewhere. Captain Harry R. Trick, M. D., delivered the address to the Medical Department in the evening. The freshman medical class numbers about seventy matriculants. Peculiarly gratifying was the registration for the new course in arts and sciences—thirty-five. It is understood that not all of these registering in this course have done so to fulfill the higher requirements for medical matriculation in force after this year, but that a considerable number have availed themselves of the course on its merits, as a means of securing a higher education for its own sake. We venture the prediction that the students will force the university authorities not only to continue but to extend this course until a fully equipped collegiate department is secured, and we are confident, not only that the authorities are more than willing to have such a suasion applied, but that they will ultimately succeed in securing the necessary means of establishing a college in Buffalo. There is an abundance of material wealth in the city, sufficiently tainted to be appropriate for an educational foundation.

